# Evaluating the impact of experience value promotes user voice toward social media: Value co-creation perspective

**DOI:** 10.3389/fpsyg.2022.969511

**Published:** 2022-08-29

**Authors:** Wanying Zhu, Zhounan Huangfu, Di Xu, Xiuping Wang, Ziang Yang

**Affiliations:** ^1^Design College, Zhoukou Normal University, Zhoukou, China; ^2^Faculty of Education, Valaya Alongkorn Rajabhat University, Pathum Thani, Thailand; ^3^Hangzhou Fourth Machinery School, E-Commerce Teaching and Research Group of Academic Affairs Office, Hangzhou, China; ^4^School of Art & Design, Zhejiang Sci-Tech University, Hangzhou, China; ^5^Adam Smith Business School, University of Glasgow, Glasgow, United Kingdom

**Keywords:** value co-creation, social value, entertainment value, information value, user loyalty, customer voice

## Abstract

Experience value is positively associated with user voice toward social media, but existing research lacks an examination of its mechanisms of action. Based on value co-creation theory, this paper explores the relationship between experience value (i.e., social value, entertainment value, information value) and customer voice, and explains the specific influence mechanism through the mediating role of user loyalty. The results of the empirical tests show that social value, entertainment value and information value have significant effects on user loyalty; user loyalty has a significant effect on promotive voice but not on prohibitive voice; user loyalty mediates the relationship between body social value, entertainment value, information value and promotive voice. The findings of this research reveal the important role of experience value on customer voice, which is an important guide for social media to achieve sustainable development.

## Introduction

With the development of technology and economy, social media is becoming an integral part of the public’s life (Yang et al., 2021a). The role of users in social media is also undergoing a profound transformation (Wang et al., 2021; Yang et al., 2022). They are becoming equal subjects of social media (Yang et al., 2021b), building personalized experience value through continuous dialogue and interaction, and jointly creating value for each other and themselves (Zhang et al., 2020). Users have become a new source of building a competitive advantage in social media in addition to suppliers, investors, and employees. Research has identified co-creating value with customers as a new strategic orientation for firms to build new strategic capital and shape new core competencies (Zhang et al., 2015; Ferm and Thaichon, 2021). In this context, it is important to promote positive interaction between users and social media, improve the quality of social media services, better meet users’ personalized needs, realize the co-creation of value between users and social media, and thus gain user loyalty (Hussain et al., 2022). And intrinsically motivate and promote users’ customer voice to social media (Ge and Gretzel, 2018), has become a hot topic of interest for academia and business. To this end, based on existing relevant studies, this study examines the potential intermediate mechanisms through which experience value affects customer voice from the perspective of value co-creation theory and provides relevant empirical research support (Kim and Choi, 2019; Namisango et al., 2021b).

According to the claims of value co-creation theory (Yi and Gong, 2013; Hsieh, 2015; Agrawal and Rahman, 2019), user loyalty is a potential intermediate mechanism by which experience value influences the effect of customer promotive voice and prohibitive voice, helping to explain the role of experience value in influencing value co-creation outcomes such as customer reviews or feedback from users. Social media has become a representative virtual place for value co-creation between the platform and users and between users and users. With the help of this platform, social media implement marketing, users share their experiences and express their ideas, and through various forms of interaction between social media and users and between users and users, users obtain value they need and social media obtain loyal users to achieve their own sustainable development. For users, the purpose and ultimate demand of value co-creation is to obtain the value they need; for social media, in order to achieve their own sustainable development, social media must provide value to users or co-creating value they need with users, so as to achieve their ultimate purpose. That is, to promote the users to feel the value from the services provided by social media, and thus to be loyal to social media (Lemke et al., 2011), and to provide suggestions for the development of social media.

The value co-creation theory based on user experience (Prahalad and Ramaswamy, 2004) believes that the interactive behavior of value co-creation between enterprises and users generates user experience, and user experience value exists in personalized user experience, and short video platform is an important place to realize value co-creation due to interactive behavior. Undoubtedly, experience value not only contributes to the revenue gained from the interactive behavior of value co-creation by users in the social media, but also is the antecedent variable that drives the social media to gain competitive advantage. Loyal users are the prerequisite for the continuous development of social media, and user loyalty has become the most basic, direct and reliable intangible asset of social media. The study of user loyalty has been a hot issue in academia, and according to previous literature (Wang et al., 2019) on the proposition that loyal users will suggest the development of social media, customer reviews or feedback, through the voice of the customer helps to provide a solid foundation and reference value for social media to improve themselves.

In summary, experience value is an important factor influencing customer voice behavior and is important for the improvement of social media. social media is a typical platform for value co-creation, and studies exploring the impact of experience value on loyalty based on value co-creation theory have emerged in the existing literature, but empirical studies on the impact of user loyalty-mediated user experience value on customer voice are more limited. So, how does the experience value according to value co-creation theory drive customer voice in the social media context? Based on this point, in order to explore the intermediate mechanism through which experience value affects customer voice, this study takes social media as the research object and examines how experience value affects customer voice through the potential mechanism of user loyalty. This paper focuses on the indirect impact of user loyalty on customer voice, and provides theoretical guidance for marketing practice and business innovation of social media.

## Theoretical background and hypotheses development

### Theoretical background

The idea of value co-creation has been widely followed and discussed in academic as well as business circles. Representative were Vargo and Lusch (2004, 2008) and Prahalad and Ramaswamy (2004) who proposed the value co-creation perspective. In other words, co-creation of value is neither a means to please the user nor an initiative of the user to create value for the company, but a process in which the user and the company create value for each other as peers. In the process of co-creation of value, both parties work together to improve the personalized service experience through continuous dialogue and interaction (Boyle, 2007; Lanier and Hampton, 2008; Svensson and Grönroos, 2008). Thus, scholars have proposed a new perspective from the user’s point of view that user value is a value created jointly between users and companies, and users and users based on interaction (Schau et al., 2009).

Social mediaserves as a representative place for value co-creation. As the initiator of value co-creation, the main goal of the user’s interactive behavior of value co-creation is to obtain the experience value. In the existing studies, there is still a lack of uniform cognition about the composition of social media users’ co-creation value. From the perspective of influencing the results of value co-creation role, scholars generally consider the following core dimensions as representative views. Proposed two-dimensional value co-creation, including information value/physical value and hedonic value. Sicilia and Palazan (2008) proposed a three-dimensional co-creation value consisting of functional value, social value, and entertainment value/emotional value. Wei (2013) proposed that co-creation value mainly includes functional value, emotional value, intellectual value and social value, i.e., four-dimensional co-creation value. Jin (2007) proposed five-dimensional co-creation value, i.e., co-creation value is composed of information value, financial value, social value, image value and entertainment value.

To sum up, different scholars have different focuses of research, and the division of individual experience value dimensions is also different. Whether it is two-dimensional experience value or multidimensional experience value, the basic principles of dimension division and measurement were divided from the value felt by users. For example, functional value, information value and utility value, intellectual value, emotional value, entertainment value and enjoyment value.

Specifically, the information value dimension and the affective dimension are the two most prevalent dimensions in the literature. The information value dimension refers to the value of knowledge derived from co-creative behaviors, while the social value dimension refers to the value of the user’s feelings or emotional characteristics during the activity. In addition, entertainment value is the direct personal satisfaction to users from the entertainment, emotional, social and other perceptible benefits generated by co-creation activities, while utility value stems from the successful completion of co-creation activities. Therefore, the research on experience value theory guided by the framework of value co-creation theory has some commonalities. Regardless of two-dimensional value experience or multi-dimensional theory multi-dimensional value experience, all of them fully consider the social attributes and emotional needs of users, and refine three dimensions of social attributes, functional attributes and entertainment attributes.

Given that the object of this study is social media, as a typical value co-creation site, users’ various value co-creation activities on social media not only enable them to obtain information value such as knowledge and information they need, but also obtain social value by means of building interpersonal relationships, communicating information and exchanging feelings in virtual situations, and for users who satisfy curiosity and seek excitement, they can also obtain entertainment value. Therefore, this study classifies the value of user experience into social value, entertainment value and information value based on the commonality of value co-creation and combined with the characteristics of social media.

### Hypotheses development

#### Experience value and user loyalty

Social media is a virtual platform with the theme of value co-creation (Kim and Choi, 2019), and user loyalty is formed when users experience value driven by value co-creation. User loyalty is a commitment that, unless there are other external factors (e.g., environmental changes, marketing strategies, etc.), loyal users will develop attitudinal preferences when using a product or service (Choi, 2015). By Rezaei et al. (2021), the quality of interactions between users was noted to positively influence functional, experiential, and symbolic benefits to users, which in turn further contribute to user loyalty. It is evident that the value of user experience is an important factor in the formation of user loyalty in both offline service industries and virtual environments (Rashid et al., 2019; Ravazzani and Hazee, 2022). Undoubtedly, with social media is used as a representative place for value co-creation. Through proactive interaction in value co-creation, users are able to obtain “social, entertaining, and informative” experiences, which in turn satisfy their experience value. In return and as a reward, experience value increases user loyalty to social media. And the higher the experience value, the stronger the loyalty of users (Chen et al., 2021).

Social value is the core demand and embodiment of user participation and interaction on the social media, and socializing through the short video platform becomes easy because it is not restricted by time, space and location. Because of the shared value base among users (Cheung et al., 2021a), social behaviors around specific topics further strengthen interactions among users and form support, friendship, and intimacy on the social media. This acquisition of social value further motivates users to be loyal to the social media (Chatterjee and Nguyen, 2021). Therefore, the social value that users co-create in social media can directly affect their loyalty to social media.

Social media are entertaining virtual places that are based on interactions between users and consist of specific, non-geographic relationships (Zadeh et al., 2019). Positive entertainment experiences inevitably lead users to acquire entertainment value. For example, posting funny short videos and sharing happy things through social media. The positive influence of users’ entertainment experience in social media usage contexts on user loyalty has also been confirmed (Bu et al., 2022). Used Tik Tok as a research object and found that user entertainment value has a positive and significant effect on social media loyalty through a structural equation modeling study. Clearly, the entertainment value in social media usage contexts also promotes user loyalty to social media.

The impact of the value of information acquired by users in social media usage contexts on user loyalty has been validated. For example, the results of an empirical study by Kao et al. (2016) showed that the value of user-acquired information positively and significantly influences loyalty through satisfaction. Studies have shown that social media has gradually become an important place to develop loyal users, and the value of user information is one of the important drivers that stimulate loyalty formation (Cheung et al., 2021b). It follows that the value of information co-created by users in social media indirectly affects their loyalty to social media. The user’s access to information value in the social media usage context is the main condition leading to satisfaction. Reviewing many related studies, most scholars agree that satisfaction and loyalty were positively correlated. Obviously, users show their loyalty to a social networking site when they get the value of information on that site or in communication with other members. Based on the above analysis, the following hypothesis is proposed.

H1a: Social value is positively associated with user loyalty.

H1b: Entertainment value is positively associated with user loyalty.

H1c: Information value is positively associated with user loyalty.

#### User loyalty and customer voice

Some social media have loyal users before they use the brand, and these user loyalty comes from word-of-mouth and promotion by other users who have used the brand before; some social media gradually acquire user loyalty through the platform after they have used it, and these user loyalty comes from their own experience and feelings (Namisango et al., 2021a). These users loyalty comes from their own experiences and feelings (Namisango et al., 2021a). For social media to be sustainable, users must ultimately become loyal to the brand. Some users become supporters of social media through the promotional voice of other users before using it; others become loyal to the social media after using it based on the combined evaluation of the social value, entertainment value and information value obtained. Social media serves as a key channel for informal marketing promotion and plays a significant role in influencing user loyalty. The use of social media to communicate brands and products influences users’ attitudes and behaviors toward brands. Customer Voice is the act of a customer spontaneously making constructive suggestions to a company to help the company’s community, products or services; Customer Voice includes the promotive voice, which emphasizes innovative ideas and suggestions, and the prohibitive voice, which refers to customers pointing out real and potential problems (Liang et al., 2012). The function of spreading brand and fostering user loyalty can only be truly realized when social media has a large number of loyal users (Tajvidi et al., 2020). Clearly, the relationship between the Customer Voice and their loyalty to social media has a reciprocal effect. Therefore, it has been suggested that social media can be used to promote the sustainability of social media by leveraging user loyalty to brands, i.e., fostering user loyalty to social media.

This reinforcement of loyalty to social media also has a positive impact on a range of behaviors such as personal contribution (Khajeheian and Ebrahimi, 2021). In addition, for impression management, customers are often willing to show their development proposals to social media, which helps to enhance the social image and identity of users in social media. User loyalty is more likely to gain users’ attention and affection for social media (Shirazi et al., 2021). Value co-creation is enhanced if social media can meet the experiential value of customers (Kennedy, 2017; Wang et al., 2019). As individuals continue to learn more about social media views and values, they are more likely to express their opinions to better promote social media (Shirazi et al., 2021). Thus, user loyalty facilitates the formation of closer relationships among users and enhances their sense of identity and belonging to social media. This continuous evolution gradually becomes a catalyst for social media contributions (Kennedy, 2017). Moreover, users will be more active in sharing their views and opinions on social media, and the higher the level of user loyalty, which contributes to a constructive voice of social media contributions. For this reason, the following hypothesis is proposed in this study.

H2a: user loyalty is positively associated with promotive voice.

H2b: user loyalty is positively associated with prohibitive voice.

#### Mediation effect of user loyalty

User loyalty has received extra attention in the field of marketing from both academics and practitioners because loyal users can bring more value to a company and help it gain a sustainable competitive advantage in a highly competitive market (Choi, 2013, 2015). Loyal users rarely switch to other products and services due to their attitudinal preferences or emotional responses to them. With the in-depth development of social media, user loyalty is seen as an intrinsic motivation and emotional immersion for users to engage in social media interactions (Ferm and Thaichon, 2021). Co-creation value is the result of the process of deep and continuous interaction between users and social media (Mladenovic and Dolenec, 2016). As a reflection of the overall feeling of customers in social media, value co-creation is an important manifestation of this positive interaction. Value co-creation contributes to the formation of ongoing social relationships, which also facilitates the formation of user-user loyalty and social relationships (Zhang et al., 2020).

The value of a good experience encourages users to be more willing to stay on social media and continue to engage in activities and contribute (Habibi et al., 2014; Zhang et al., 2015), which also provides a good basis for the occurrence of customer voice. Thus, user loyalty can predict customers’ voice behavior. High levels of social value can also better build emotional connections between users and brands or other users, deepen users’ understanding of social media, and strengthen their relationships with brands and brand communities as a whole (Kaur et al., 2016). This reinforcement of experiential value gradually increases user loyalty, enhances the sense of belonging and identification with social media (Lin et al., 2019), and ultimately provides a catalyst for a range of social media support or contribution behaviors. Thus, user loyalty mediates the relationship between social value and customer voice.

Entertainment value also has a significant positive impact on personal emotions. social media gain the attention of users by producing entertainment content, which triggers positive emotions in users (Rieger et al., 2014). This emotional stimulation and reinforcement will increase user loyalty to social media (Kaur et al., 2016), thus helping to motivate and enthuse users to contribute to social media. Entertainment value explains the level of user loyalty to social media, further contributing to positive attitudes and engagement behaviors toward social media activities (Wang et al., 2019). Thus, user loyalty mediates the relationship between entertainment value and customer voice.

The value of information is closely related to the formation of user loyalty, and user loyalty may also contribute to the creation of a customer voice. On the one hand, the value of information encourages users to share their knowledge on social media and also enhances their sense of belonging to social media, thus helping to promote user loyalty (Wang et al., 2019). On the other hand, when users perceive the value and significance of social media and develop strong loyalty, they are more willing to contribute more to the development of social media (Lin et al., 2019), which may provide an important prerequisite for the voice of customers in social media. Therefore, the relationship between user loyalty mediated information value and customer promotion voice. In summary, the following hypotheses are proposed in this study.

H3a: user loyalty plays a mediating role between social value and promotive voice.

H3b: user loyalty plays a mediating role between social value and prohibitive voice.

H4a: user loyalty plays a mediating role between entertainment value and promotive voice.

H4b: user loyalty plays a mediating role between entertainment value and prohibitive voice.

H5a: user loyalty plays a mediating role between information value and promotive voice.

H5b: user loyalty plays a mediating role between information value and prohibitive voice.

**FIGURE 1:**
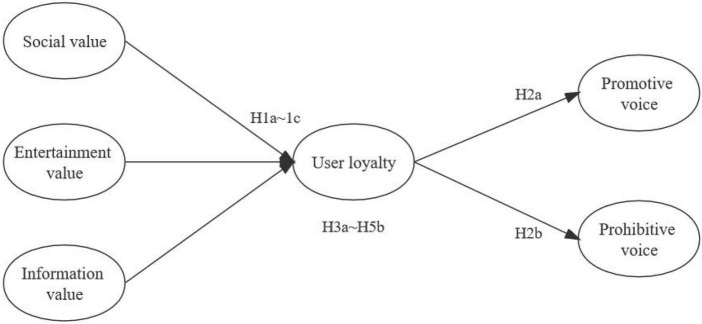
Theoretical model.

In summary, this study proposes a research model as shown in Figure 1.

## Method

### Participants and procedure

In this study, Tik Tok respondents were selected as participants. Tik Tok was founded in May 2017. As of December 23, 2021, Tik Tok is the most visited short-form social video platform in the world, with respondents in countries and regions such as the United States, Germany, Japan, Paris, Dubai, Singapore, Jakarta, and Seoul. Obviously, the selection of Tik Tok as the sample survey participants has the representativeness of the questionnaire.

The data collection for this study was mainly done by questionnaire method. Questionnaires were distributed in the form of paper-based questionnaires. The questionnaire was distributed over a 5-month period from November 2021 to March 2022. A total of 550 questionnaires were distributed, 502 questionnaires were collected, 58 invalid questionnaires were excluded (including 6 questionnaires with all or almost all answers checked, 6 questionnaires with too short a response time, and 5 questionnaires with obvious logical errors), and 481 valid questionnaires were finally obtained, with a return rate of 91.3%. From the effective sample, there are more men than women; the age distribution is mainly young, with 86.3% of respondents under 39 years old; the proportion of respondents with education of bachelor or above is 90.9%. Details of the sample are as follows. There were 250 (52%) males and 231 (48%) females, with an equal ratio of males to females. Among them, 318 were married, 159 were unmarried, and 4 were divorce. Less than 6 months (12 respondents) 2.5%; 6 months∼1 year (59 respondents) accounted for 12.3%; 1 year∼2 years (204 respondents) accounted for 42.4%; more than 3 years (206 respondents) accounted for 42.8%. In terms of age 18 or less respondents are the least, accounting for 2.1%. 30∼39 respondents are the most, accounting for 44.5%. 19∼29 respondents account for 39.47%. 40∼49 respondents account for 9.8%. 50 or above respondents account for 4.0%. In terms of occupational origin, in descending order, they are as follows. Civil servant with 43.51% (209 respondents); executive in private enterprise with 31.41% (151 respondents); student with 11.91% (57 respondents); freelance with 6.91% (33 respondents); clerk in state owned enterprise accounted for 5.61% (27 respondents); executive in private enterprise accounted for 0.81% (4 respondents). In terms of education, the situation is as follows. College and blow 44 (9.1%); undergraduate 60 respondents (21.6%); master’s degree and above 377 respondents (78.4%). In terms of consumption level, below 2000 (RMB) accounted for 7.1% (34 respondents); 2000∼3999 (RMB) accounted for 15.4% (74 respondents); 4000∼5999 (RMB) accounted for 21.8% (105 respondents); 6000 or more (RMB) accounted for 55.7% (268 respondents).

### Measures

There are six variables in this study: social value, entertainment value, information value, user loyalty, promotive voice and prohibitive voice. The variables in this study were measured according to scales that have been shown to be reliable in previous studies. Specifically, social value and entertainment value were measured using Sweeney and Soutar (2001) scale, information value was measured according to Jin (2007); Papacharissi and Rubin (2000), and user loyalty measured according to the studies of Kim and Son (2009); Choi (2015) and Wang et al. (2021). In addition, promotive voice and prohibitive voice Liang et al. (2012) used the measurement questions developed by Liang et al. (2012). This study was used to describe the measurement tools used in this study, using the Likert scale (1 = Strongly disagree, 7 = Strongly agree). The question items of the variable measurements and their references are shown in Table 1.

**TABLE 1:** Variables and measurement item.

Variables	Items	Sources
Social value	SV1. Tik Tok provides a lot of useful information on the purchase, use and maintenance of products	Sweeney and Soutar (2001)
	SV2. Tik Tok’s users often share their experiences and tips with each other	
	SV3. Some of Tik Tok’s users have real-life contacts and maintain relationships	
Entertainment value	EV1. The activities on Tik Tok and the communication with others always make me feel happy	Sweeney and Soutar (2001)
	EV2. posting opinions, discussing and communicating on Tik Tok is fun in itself	
	EV3. Participating in Tik Tok has relieved me of the stress of work and life, and I can get a sense of relaxation	
	EV4. When I feel bored, I will use Tik Tok to kill time	
Information value	IV1. Tik Tok has provided a lot of useful information	Jin (2007); Papacharissi and Rubin (2000)
	IV2. I benefit a lot from the experiences of other users on Tik Tok	
	IV3. I can get the information and materials I need from Tik Tok when I need them	
User loyalty	UL1. I consider myself to be a very loyal Tik Tok user	Kim and Son (2009); Choi (2015), Wang et al. (2021)
	UL2. I like Tik Tok very much	
	UL3. Feel I am committed to Tik Tok	
	UL4. In general, I am very loyal to Tik Tok	
Promotive voice	PMV1. I will make suggestions to help Tik Tok improve product quality	Liang et al. (2012)
	PMV2. I will make suggestions to improve the service in Tik Tok	
	PMV3. I will make suggestionsto help Tik Tok develop marketing plans	
	PMV4. I will put forward my suggestions for improving Tik Tok	
Prohibitive voice	PHV1. I will point out the problems of services to Tik Tok	Liang et al. (2012)
	PHV2. I will point out the problems of products to Tik Tok	
	PHV3. I would like to criticize Tik Tok and help it improve	
	PHV4. I would like to express opinions on the problems in Tik Tok	

### Data analysis method

This study uses structural equation modeling (SEM) to analyze value promotes user voice toward social media. Structural equation modeling has significant advantages in analyzing the hypothesized relationships between variables, not only to test the basic hypothesis well, but also to test the mediating utility accurately. Partial least squares structural equation modeling (PLS-SEM) has gained considerable attention in recent years in a variety of disciplines, including marketing, strategic management, operations management, and organizational behavior. PLS is a composite-based SEM method that aims to maximize the interpretable variance of construct-dependent ideas in path models. In contrast to other SEM techniques, PLS allows researchers to simultaneously estimate complex interrelationships involving multiple constructs and indicators and their direct, indirect, or moderating relationships. Therefore, structural equation modeling is commonly used in empirical studies. For these reasons, this study applied the Smart PLS to analyze the collected data for structural equation modeling.

## Data analysis and results

### Outer model analysis

In the outer model analysis, outer model primarily report Factor Loading, Cronbach’s alpha, Composite Reliability (CR), and Average Variance Extracted (AVE) for all dimensions (Kline, 2011). Outer model was evaluated and corrected. If the factor loadings > 0.50, Cronbach’s alpha > 0.70, CR > 0.60, and AVE > 0.50 means that the outer model has good convergent validity (Fornell and Larcker, 1981; Nunnally and Bernstein, 1994). Outer model is shown in Table 2. Factor loadings of all dimensions are between 0.714 and 0.936, Cronbach’s alpha is between 0.703 and 0.941, CR is between 0.834 and 0.949, and AVE is between 0.610 and 0.822. The above analysis shows that the measurement model of this study has good convergent validity.

**TABLE 2:** Outer model analysis.

Construct	Item	Factor Loadings (*t*-value)	Cronbach’s alpha	CR	AVE
Social value (SV)	SV1	0.834	0.762	0.861	0.674
	SV2	0.857			
	SV3	0.768			
Entertainment value (EV)	EV1	0.775	0.824	0.883	0.654
	EV2	0.809			
	EV3	0.837			
	EV4	0.813			
Information value (IV)	IV1	0.770	0.703	0.834	0.626
	IV2	0.794			
	IV3	0.810			
User loyalty (UL)	UL1	0.762	0.838	0.891	0.673
	UL2	0.785			
	UL3	0.866			
	UL4	0.863			
Promotive voice (PMV)	PMV1	0.766	0.787	0.862	0.610
	PMV2	0.819			
	PM3	0.821			
	PMV4	0.714			
Prohibitive voice (PHV)	PHV1	0.935	0.941	0.949	0.822
	PHV2	0.820			
	PHV3	0.931			
	PHV4	0.935			

In this study, discriminant validity was analyzed based on the claims of Henseler et al. (2015), Henseler et al. (2016) and Hair et al. (2019). Specifically, this study calculates the heterotrait-monotrait (HTMT) correlation ratio to calculate the discriminant validity of the measurement model. HTMT is the average of the Heterotrait-Heteromethod correlations relative to the average of the Monotrait-Heteromethod correlations. The study states that the HTMT should be less than 1, and better if it is less than 0.85, then it indicates that there is a distinction between the two constructs that is different from each other, i.e., there is no cross-factors. Results of discriminant validity by HTMT is shown in Table 3. The results of the data analysis analysis revealed HTMT ratios < 0.85 for each pair; this indicates good discriminant validity of the measurement model in this study.

**TABLE 3:** Results of discriminant validity by HTMT.

	Entertainment value	Information value	Prohibitive voice	Promotive voice	Social value
Information value	0.481				
Prohibitive voice	0.150	0.248			
Promotive voice	0.465	0.536	0.217		
Social value	0.549	0.544	0.132	0.369	
User loyalty	0.664	0.555	0.065	0.552	0.528

### Inner model analysis

The path coefficients are shown in Table 4. Social value (SV; β = 0.169, *p*-value < 0.001), entertainment value (EV; β = 0.402, *p*-value < 0.01) and information value (IV; β = 0.217, *p*-value < 0.001) are positively associated with user loyalty (UL). Therefore, H1a, H1b and H1c are significant. User loyalty (UL; β = 0.459, *p*-value < 0.001) is positively associated with promotive voice (PMV). Therefore, H2a is significant. User loyalty (UL; β = 0.063, *p*-value > 0.001) isn’t positively associated with prohibitive voice (PHV). Therefore, H2b isn’t significant.

**TABLE 4:** Regression coefficient.

	Path coefficient (β)	Standard deviation (STDEV)	T Statistics (| O/STDEV|)	*p*-value	Result
H1a: SV->UL	0.169	0.047	3.621	***	Significant
H1b: EV->UL	0.402	0.057	7.000	***	Significant
H1c: IV->UL	0.217	0.044	4.950	***	Significant
H2a: UL->PMV	0.459	0.041	11.170	***	Significant
H2b: UL->PHV	0.063	0.088	0.473	ns	Not significant

***p-value < 0.001, ns, non-significant.

The results of the mediating effect analysis are shown in Table 5. This study analyzes the mediating effect according to Zhao et al. (2010). The above analysis of regression coefficients shows that H2b is not significant. Therefore, this study only needs to analyze the mediating effect of user loyalty between social value, entertainment value, information value and promotive voice.

**TABLE 5:** The analysis of mediating effect.

Effect	Regression weight	Standard deviation	*t*-value	Bias corrected 95% lower bound	Bias corrected 95% upper bound
Indirect effect: SV → UL → PMV	0.078	0.023	3.395	0.037	0.125
Indirect effect: EE → UL → PMV	0.185	0.031	5.911	0.122	0.249
Indirect effect: IV → UL → PMV	0.100	0.021	4.759	0.062	0.144

SV, social value; EV, entertainment value; IV, information value; UL, user loyalty; PMV, promotive voice; PHV, prohibitive voice.

In the relationship between user loyalty mediated social value and promotive voice. The analysis of the indirect effect data is as follows. Regression weight = 0.078, *t*-value = 3.395 > 1.96, Bias corrected 95% lower bound = 0.037 and Bias corrected 95% upper bound = 0.125. Apparently, Bias corrected 95% does not contain 0. Therefore, H3a is significant. In the relationship between user loyalty-mediated entertainment value and promotive voice. The analysis of the indirect effect data is as follows. Regression weight = 0.185, *t*-value = 5.911 > 1.96, Bias corrected 95% lower bound = 0.122 and Bias corrected 95% upper bound = 0.249. Apparently, Bias corrected 95% does not contain 0. Therefore, H4a is significant. In the relationship between user loyalty-mediated information value and promotive voice. The analysis of the indirect effect data is as follows. Regression weight = 0.100, *t*-value = 4.759 > 1.96, Bias corrected 95% lower bound = 0.062 and Bias corrected 95% upper bound = 0.144. Apparently, Bias corrected 95% does not contain 0. Therefore, H5a is significant.

## Research results and discussion

### Findings and discussion

First, the results of the data analysis showed that the three dimensions of experience value have a positive effect on user loyalty. The data test results are consistent with existing studies. The interaction between users through social media for resource integration influences co-creation value, which is the most important influencing factor of social media co-creation value. The reason for this is that social media is a virtual place established by users based on their interests and hobbies, and has the characteristics of easy communication and openness. These features enable users to interact with each other in social media, and then realize the process of resource integration such as social value, entertainment value, information value and other social relationship integration. By social media users identifying with the emotional resources shared by other users, as well as expanding their friendships, etc., they can bring pleasure and mental enjoyment to other users, thus creating loyalty to social media. At the same time, through the interaction between customers and users, the interest themes of social media and good service information are better disseminated in the process of resource integration of customers, effectively enhancing users’ trust in social platforms and brand image recognition, thus creating loyalty to social media. In addition, through resource integration, social media users can more fully access and use the value of information they need, thus creating loyalty to social media.

Second, the results of structural equation modeling analysis indicate that user loyalty has a significant effect on user loyaltycustomer promotive voice and a negative effect on prohibitive voice. The results of hypothesis testing are consistent with the results of existing studies and logical advancement. It is speculated that the reason may be that, under the influence of co-creation value, experience value influences the formation of user loyalty to social media through resource integration, and user loyalty is the main influencing factor reflecting social media customer voice. The experience value generated through value co-creation activities not only helps social media to provide users with effective service information; but also helps users to provide reasonable suggestions and feedback to social media. Experience value can enhance user loyalty to social media, and help users accept social media promotion and recommendation activities. Under the role of loyalty, the integration of information resources such as information sharing, usage suggestions and feedback can be achieved smoothly. Through resource integration, users can grasp comprehensive social media service information, more smoothly use social media for interactive communication and resource sharing, effectively promote the user’s demand in terms of information value and entertainment value. And through the integration of social media resources can promote the user’s in-depth understanding of social media, through the use of platform services to meet their own needs, and then enhance the user’s loyalty to social media, and effectively promote customer promotion voice. In other words, through value co-creation activities and social media-based resource integration, user loyalty mediates the relationship between experience value and customer promotive voice.

Third, user loyalty has a mediating role in the relationship between experience value and promotive voice. The social value generated by users’ value co-creation interaction in the short video platform can promote users’ loyalty to the short video platform, which in turn influences users to social media promotional voice, rather than prohibitive voice, through loyalty. User loyalty plays a mediating role between entertainment value and promotive voice. This study takes users of Tik Tok as the research object. In terms of user performance, the number of likes, comments, retweets and downloads of works posted on Tik Tok is much higher than those posted by users. This reflects that value co-creation activities through short video platforms are an important source of user experience value and an important path for user experience value to influence customer voice. The acquisition of entertainment value is the most important motivating factor for users to use social media and perform interactive behaviors. After becoming a social media user, a user without user loyalty acquires entertainment value through social media, which first drives user loyalty and then drives promotional voice, rather than prohibitive voice, toward the social media. User loyalty plays a mediating role between information value and promotive voice. This suggests that access to information value is an important driver of not only user loyalty formation, but also an important influencing factor in the formation of customer voice. Although information value and social value are also acquired through social media, information value not only enables user loyalty to social media, but also further influences users’ promotive voice.

### Theoretical contributions

First, this study explores the impact of user loyalty on the experience value obtained through users’ interaction behavior from the perspective of value co-creation, which enriches the research results of value co-creation theory. The results of the study show that social value, entertainment value and information value all have a positive impact on user loyalty, and uncover the key drivers of social media to create a customer promotive voice and maintain the sustainability of the platform. Social media is a value co-creation vehicle, and the combined effect of experience value and user loyalty drives the sustainability of social media development and platform building.

Secondly, this study constructs the mechanism of the role of experience value in influencing customer voice. In previous studies on social media user loyalty, scholars explored the antecedents of experience value or user loyalty, respectively, and did not further discuss the relationship between the role of user loyalty on customer voice. In contrast, customer promotive voice and prohibitive voice, which directly reflect the direct results of social media operational performance and are effective indicators for evaluating the results of user loyalty in social media, lack the support of empirical studies. To this end, the theoretical model constructed in this study provides a new perspective on customer voice.

Third, this study examines the mechanism by which the experience value acquired by social media users influences customer voice through the mediating role of user loyalty. This study finds that user loyalty plays a mediating role between experience value and promotive voice toward social media, bridging the gap in previous studies on the antecedent influences of customer voice. This research shows that user loyalty plays a very important role in the innovative management and continuous development of social media, and the data analysis directly demonstrates the relationship between experience value, user loyalty and customer voice, and explores new ways to influence customer voice in the context of social media usage.

### Practical implications

The findings of this study have the following practical implications for companies to build, engage, manage and maintain social media to achieve competitive advantage. First of all, information value and social value are the main antecedent drivers of user loyalty. For social media operators, it is necessary to take more measures to improve users’ perception of information value and social value. In an era when short-form social media are commonly integrated into the lives of the general public, users rely more on information from other users. On the one hand, social media should create opportunities for users to interact; on the other hand, social media should guide users to actively spread and promote this information. More importantly, entertainment function of social media is given full play to highlight the status of users with high contribution in the minds of other users. Then, some of the users with higher perception of entertainment value will actively share information to improve other users’ perception of entertainment value, and then cultivate more users who are loyal to social media.

Second, cultivating users’ user loyalty is the ultimate purpose of building social media for enterprises. Social value, entertainment value and information value are important antecedent variables in the formation of user loyalty, but the formation of customer voice is the result of playing a mediating mechanism of user loyalty. In other words, user loyalty facilitates the realization of customer voice. On the one hand, platforms should enhance users’ stimulation of experience value (i.e., social value, entertainment value and information value), which in turn stimulates the formation of user loyalty. On the other hand, platforms should recognize the mediating role of user loyalty in influencing customer voice on experience value, so as to smoothly realize the transformation from non-transactional to transactional relationship between users and companies.

Third, companies should realize the importance of social media. Social media help to enhance the positive relationship between users and enterprises, and regular users who are already loyal to social media as an important component of the membership, the value of their experience gained in social media drives their suggestions to social media through user loyalty, driving the sustainable development of social media. Therefore, for social media that already have many loyal users, engaging loyal users in interactions is a core way to drive customer voice and can be an important way to form loyal users and promotive voice.

### Research limitations and future research directions

Several limitations and future works should be focus on as follows. First, the sample subjects for data collection in this study were mainly young people. Further research could consider possible behavioral differences among social media users of different ages and whether there are differences in their influence on customer voice to be further explored. Second, different types of social media were not studied in this study, and Tik Tok was the main object of analysis. Subsequent studies may consider whether there are differences in the mechanism of influencing the voice of customers of different types of social media users. Finally, in this study, the validation data of the research model are cross-sectional in nature, whereas the use of dynamic data can both test the association and reveal the causality of the model. Therefore, other methods (e.g., qualitative research) can be used to study it in the future.

## Data availability statement

The raw data supporting the conclusions of this article will be made available by the authors, without undue reservation.

## Author contributions

WZ, ZH, and DX: conceptualization and methodology, formal analysis, and investigation and visualization. All authors wrote the original draft preparation, wrote about review and editing, and have read and agreed to the published version of the manuscript.
